# The Role of Sleep on Physical and Cognitive Performance of Ultra-Endurance Athletes: A Systematic Review

**DOI:** 10.3390/jcm15041398

**Published:** 2026-02-10

**Authors:** Larissa Quintão Guilherme, Bruno Otávio Rodrigues, Carla de Oliveira Barbosa Rosa, Luciano Bernardes Leite, Volker Scheer, Pedro Forte, Helen Hermana Miranda Hermsdorff, Ana Claudia Pelissari Kravchychyn, Helton de Sá Souza

**Affiliations:** 1 Department of Physical Education, Federal University of Viçosa (UFV), Viçosa 36570-000, MG, Brazil; larissa.guilherme@ufv.br (L.Q.G.); helton.souza@ufv.br (H.d.S.S.); 2 Laboratory of Food Intake, Department of Nutrition and Health, Federal University of Viçosa (UFV), Viçosa 36570-000, MG, Brazil; bruno.o.rodrigues@ufv.br (B.O.R.); carla.rosa@ufv.br (C.d.O.B.R.); 3 Ultra Sports Science Foundation, 109 Boulevard de l’Europe, 69310 Pierre-Benite, France; volkerscheer@yahoo.com; 4 Department of Sports Sciences, Instituto Politécnico de Bragança, 5300-253 Bragança, Portugal; 5 Department of Sports, Higher Institute of Educational Sciences of the Douro, 4560-708 Penafiel, Portugal; 6 CI-ISCE, Instituto Superior de Ciências Educativas do Douro (ISCE Douro), 4560-547 Penafiel, Portugal; 7 Research Center for Active Living and Wellbeing (LiveWell), Instituto Politécnico de Bragança, 5300-253 Bragança, Portugal; 8 Laboratory of Clinical Analysis and Genomics, Department of Nutrition and Health, Federal University of Viçosa (UFV), Viçosa 36570-000, MG, Brazil; helenhermana@ufv.br (H.H.M.H.); ana.pelissari@ufv.br (A.C.P.K.); 9 Laboratory of Energy Metabolism and Body Composition, Department of Nutrition and Health, Federal University of Viçosa (UFV), Viçosa 36570-000, MG, Brazil

**Keywords:** sleep, sleep quality, athletic performance, cognitive performance, ultra-endurance

## Abstract

**Background/Objectives:** Sleep is an important factor for recovery and performance in endurance sports, yet its role in ultra-endurance events remains unclear due to extreme physical and cognitive demands and disrupted sleep patterns. This systematic review aimed to analyze the role of sleep in physical and cognitive performance in ultra-endurance athletes. **Methods:** This systematic review followed PRISMA guidelines. A comprehensive search was conducted in May 2025 across PubMed/Medline, Embase, SPORTDiscus, and Web of Science. Two researchers independently screened, selected, extracted, and assessed data quality using the JBI tools (PROSPERO ID: CRD420251042220). **Results:** Of 424 articles, 16 met inclusion criteria, totaling data from 1389 athletes. Regarding physical performance, better outcomes were associated with no or less sleep during competition (TST), extended sleep the night before, and increased time in light sleep. In contrast, longer wake time, lower sleep quality, greater sleepiness during competition, and higher sleep efficiency were linked to poorer performance. Cognitive performance was positively associated with pre-race sleep quality and mid-race naps. Conversely, greater accumulated sleep before testing was linked to worse cognitive outcomes. **Conclusions:** Sleep, particularly total sleep time (TST), plays an important role in ultra-endurance performance, although this relationship may be non-linear and influenced by race context and individual strategies. Pre-race and intra-race sleep strategies such as napping and extended sleep may benefit performance. Further rigorous and longitudinal studies are needed to clarify sleep’s impact on performance and recovery in ultra-endurance contexts.

## 1. Introduction

The practice of ultra-endurance sports has exhibited exponential growth in recent years, contributing to a significant increase in the number of events dedicated to these disciplines [1,2]. These sports are defined by prolonged physical demands, typically lasting more than six hours and involving distances greater than 42.195 km (the standard marathon distance) [3,4]. Among the most commonly practiced ultra-endurance disciplines are ultramarathon running, long-distance triathlon, swimming, cycling, cross-country skiing, and adventure racing [3,4].

These disciplines place extreme demands on the human body [1], requiring prolonged exertion under challenging physiological and environmental conditions [5]. Such demands may lead to adverse health effects for participants [6,7], as they require a high level of both physical and cognitive performance. In this context, there is growing scientific interest in understanding the factors that influence performance in ultra-endurance disciplines, such age, sex, energy balance, body composition, maximal aerobic velocity, and nutritional behavior [1,8,9].

Sleep, however, has received little attention in this field, despite its well-established role in overall health and well-being. Adequate sleep is fundamental for the restoration of both physical and cognitive functions [10,11,12,13], and insufficient sleep duration and/or poor sleep quality (SQ) have been consistently associated with negative outcomes across multiple physiological systems, including the immune, cardiovascular, and musculoskeletal systems [14,15]. Furthermore, sleep disturbances can impair cognitive functions such as mood, alertness, and reaction time [10,16,17], which may significantly compromise athletic performance [18,19].

In sports, the relevance of sleep extends beyond general health. Studies have shown that athletes frequently experience poor sleep quality and insufficient sleep duration [18,19,20], factors that can contribute to declines in physical performance, including reductions in strength, speed, and power, as well as cognitive impairments, such as memory loss, decreased attention, and impaired decision-making [10]. These consequences are particularly critical for ultra-endurance athletes, who are exposed to high physiological demands over extended periods.

To date, no studies have specifically considered sleep as a predictor of performance in ultra-endurance sports. Therefore, the present study aimed to analyze sleep-related characteristics and their associations with physical and cognitive performance in ultra-endurance athletes.

## 2. Materials and Methods

### 2.1. Protocol and Registration

This systematic review was conducted in accordance with the 2020 Preferred Reporting Items for Systematic Reviews and Meta-Analyses (PRISMA) statement [21]. The PRISMA checklist is available in Supplementary Table S1, and the study protocol was registered in the International Prospective Register of Systematic Reviews (PROSPERO) under the registration number CRD420251042220.

### 2.2. Eligibility Criteria

Study eligibility was defined according to the PECOS framework (Population, Exposure, Comparators, Outcomes, Study design), which guided the inclusion criteria (Table 1). The central research question was: “Does better SQ and quantity improve the physical or cognitive performance of ultra-endurance athletes?”

Eligible participants included ultra-endurance athletes of any gender and performance level (elite or amateur), who have participated in at least one ultra-endurance event. Eligible events encompassed running, cycling, swimming, triathlon, rowing, or skiing, whether single-stage or multi-stage, and must exceed the standard marathon distance (42.195 km) or last at least six hours. This includes off-road, track, treadmill, or road events.

The criteria for non-inclusion or exclusion were as follows: (1) endurance events with distances shorter than 42.195 km or durations of less than six hours; (2) outcomes limited exclusively to physiological or biochemical markers (e.g., cortisol, cytokines, heart rate), with no correlation to performance; (3) assessments focused solely on mood, stress, anxiety, or motivation, without any association with cognitive function or performance; (4) publications that were not full original studies, such as abstracts, letters, case series, review articles, or studies for which full-text access was unavailable and (5) studies conducted in animal models.

### 2.3. Database and Search Strategy

A systematic literature search was conducted in May 2025 independently and in parallel by two authors (LQG and BOR), using four major databases: PubMed/Medline, Embase, SPORTDiscus, and Web of Science. No restrictions were applied regarding publication date or language during the identification and selection process. Search descriptors were selected based on terms from Medical Subject Headings (MeSH–PubMed), Health Sciences Descriptors (DeCS), and Emtree (Embase), all in English.

Three sets of indexed terms were used (1—population, 2—exposure, and 3—outcome), searched across all fields and combined using the Boolean operators “OR” and “AND” to appropriately link the search terms.

Set 1 included terms related to ultra-endurance sports: (“ultra-endurance running” OR “ultra marathon” OR “ultramarathon running” OR “ultra-endurance” OR “ultra-athlete” OR “ultra-endurance training” OR “ultra-distance” OR “ultramarathon” OR “ultra-event” OR “trail run” OR “mountain run” OR “ultra run” OR “ultra trail” OR “ultra endurance” OR “cross country skiing” OR “cross country ski” OR “cross country skiers” OR “skating” OR “ironman” OR “triathlon” OR “bicycling” OR “ultraendurance sports” OR “treadmill exercise” OR “high intensity exercise”).

Set 2 included different terminologies related to sleep: (“sleep” OR “sleeping habits” OR “sleep habits” OR “sleep duration” OR “sleep hygiene” OR “sleep quality” OR “sleep latency” OR “sleep stage” OR “sleep stages” OR “sleepiness” OR “sleep problem” OR “sleep disorder” OR “sleep–wake disorders” OR “sleep deprivation” OR “insufficient sleep” OR “sleep fragmentation” OR “fragmented sleep” OR “sleep loss” OR “restricted sleep” OR “sleep restriction” OR “sleep time” OR “night sleep” OR “sleep pattern” OR “sleep patterns” OR “somnolence” OR “sleep debt” OR “sleep spindle” OR “sleep spindles”)

And set 3 included terms related to performance as the primary outcome: (“Physical Functional Performance” OR “Athletic Performance” OR “Physical performance” OR “Psychomotor Performance” OR “Cognitive performance” OR “Time to complete the race” OR “Final ranking” OR “Placement” OR “Maximal oxygen uptake” OR “VO_2_max” OR “Muscular power” OR “Time to exhaustion” OR “Power output” OR “Average speed” OR “Distance covered” OR “Reaction Time” OR “Response Speed” OR “Response Time” OR “Response Times” OR “Decision-Making” OR “Executive Function” OR “Executive Functions” OR “Cognitive Flexibility” OR “Cognitive Flexibilities” OR “Attention” OR “Focus of Attention” OR “Attention Focus” OR “Decision-making ability” OR “Mental confusion” OR “Working memory”). The complete search strategy for each database is presented in Supplementary Table S2.

### 2.4. Data Extraction and Selection Process

Initially, all retrieved records were imported into Zotero software (version 7.0.15; Corporation for Digital Scholarship, Vienna, Austria), which assists in organizing references and identifying duplicate entries for subsequent removal [22].

Following the removal of duplicates, the remaining records were imported into the Rayyan QCRY^®^ platform (Qatar Computing Research Institute, Doha, Qatar) [23], where we screened the papers based on title and abstract review, followed by full-text reading to assess the eligibility of the studies.

All procedures were carried out independently, in parallel, and in a blinded manner by two reviewers (LQG and BOR) to ensure greater reliability in the process. In cases of disagreement during the title and abstract screening phase, the study was automatically included for full-text review. When discrepancies arose after full-text reading, they were resolved by consensus between the two reviewers (LQG and BOR), or, if consensus was not reached, by consulting a third reviewer (HdSS).

After the full screening of studies, data extraction was performed using a summary table created by the authors (LQG and BOR). This table included the following relevant information: (1) study reference (author and year of publication); (2) study design; (3) sample characteristics (number of participants, sex, age); (4) exposure characteristics (sleep-related variables and sleep assessment methods); (5) characteristics of the ultra-endurance sport (ultramarathon, triathlon, cycling, swimming, rowing, skiing); (6) performance variables assessed (physical or cognitive, along with the methods used to assess performance); and (7) main findings.

Data extraction was conducted using a standardized form in Microsoft^®^ Excel^®^ 2019 (version 16.0; Microsoft Corp., Redmond, WA, USA), which contained the variables of interest (Table 2). Data were independently extracted by the authors (LQG and BOR) and subsequently verified for consistency. Any discrepancies were resolved by a third author (HdSS).

### 2.5. Study Quality Assessment

**Figure 1:**
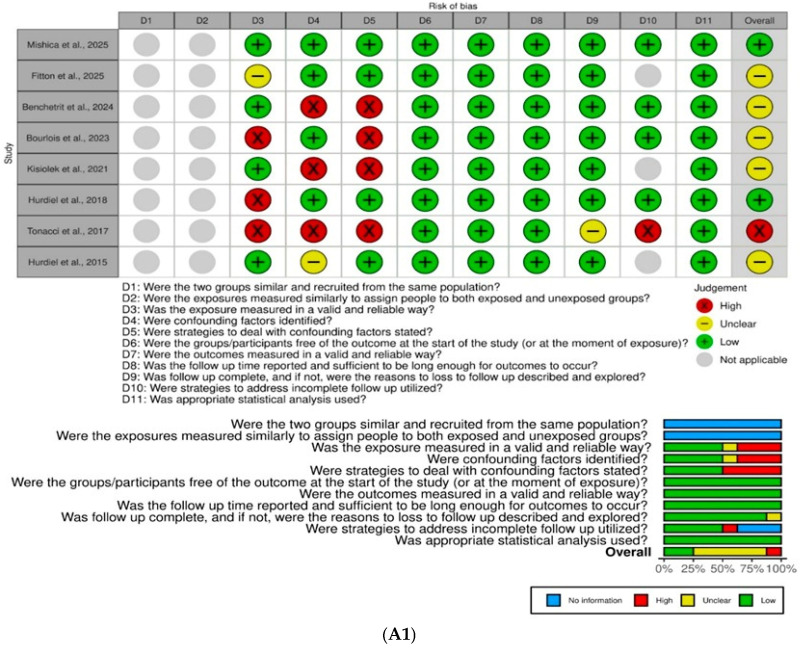

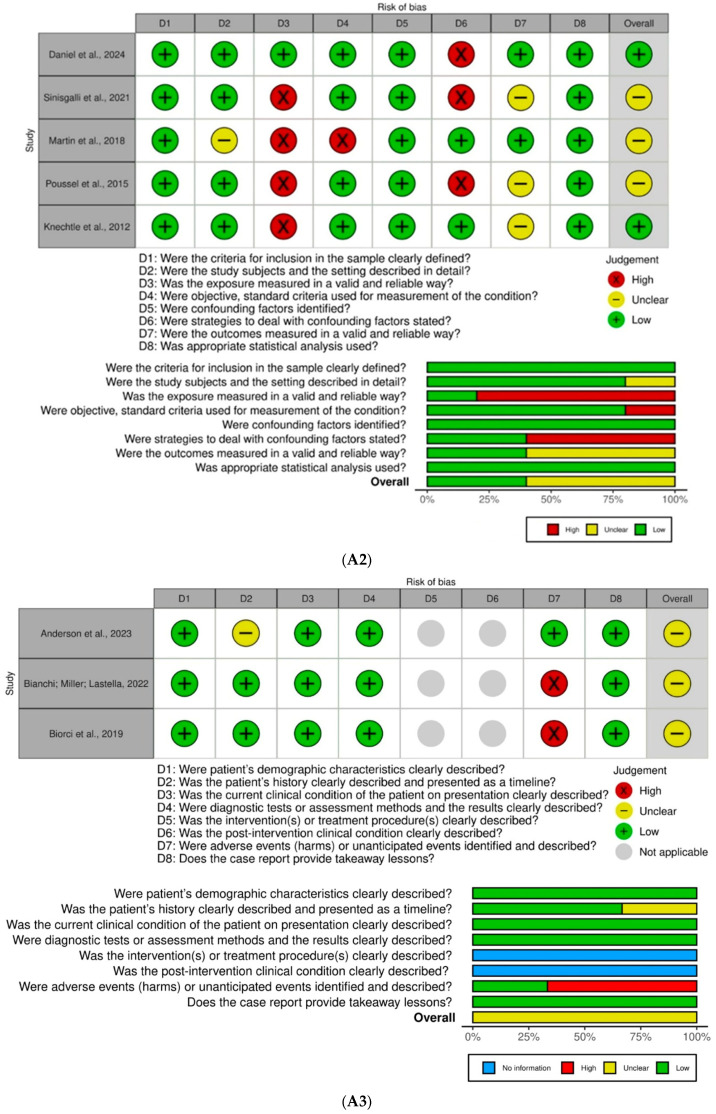
Risk of bias assessment according to the Joanna Briggs Institute: (**A1**) Cohort Studies, (**A2**) Analytical Cross-Sectional Studies, and (**A3**) Case Reports [27,28,29,30,31,32,33,34,35,36,37,38,39,40,41,42].

The risk of bias assessment was performed independently and in parallel by two researchers (LQG and BOR), with any discrepancies resolved by consensus. This critical appraisal was conducted using the Joanna Briggs Institute (JBI) Reviewer’s Manual (Joanna Briggs Institute, Adelaide, SA, Australia) and its specific Critical Appraisal Tools tailored to each study design [24] (Figure 1).

Three JBI checklists were applied according to the design of the studies evaluated: the Checklist for Cohort Studies, which includes 11 items; the Checklist for Analytical Cross-Sectional Studies, containing 8 items; and the Checklist for Case Reports, also comprising 8 items. Each item was assessed as “met = yes,” “not met = no,” or “unclear,” and in some cases, as “not applicable.”

The JBI manual recommends that authors predefined criteria to classify the risk of bias level for each study, since the tool does not provide a standardized scoring system to determine whether the risk of bias is low, moderate, or high for each article (JBI Manual for Evidence Synthesis, 2024) [25]. To classify each article individually, therefore, the following cut-off points were adopted according to the percentage of affirmative responses: low (≥70%), moderate (between 50 and 70%) and high risk of bias (<50%) [26].

### 2.6. Risk of Bias

The assessment of risk of bias is summarized in Figure 1, according to the type of study evaluated. Figure 1A1 presents the analysis of the eight prospective studies: two were classified as having low risk of bias, five as moderate risk, and one as high risk. The studies classified as moderate risk frequently relied on subjective or non-validated methods to assess sleep exposure and did not adequately address potential confounding factors. The only study rated as high risk [27] failed to provide sufficient follow-up and did not report strategies to deal with incomplete data, substantially increasing its overall risk.

Figure 1A2 displays an assessment of the five cross-sectional studies: two were judged to have low risk of bias, and three moderate risk. The main reasons for moderate risk in this group included the use of non-validated sleep measures, lack of standardized outcome criteria, and insufficient reporting of strategies to control for confounding factors. For instance, Sinisgalli et al. [28] and Poussel et al. [29] both showed high risk in the validity of outcome measurement, while Martin et al. [30] did not address confounders in the analysis.

Figure 1A3 shows the analysis of the three case reports, all of which were classified as having moderate risk of bias. In these studies, the domain most commonly associated with increased bias was the lack of identification or reporting of adverse events (harms) or unexpected outcomes. Specifically, Anderson et al. [31] also presented unclear reporting of patient history, while Bianchi et al. [32] and Biorci et al. [33] did not describe harms, contributing to their moderate risk classification.

## 3. Results

### 3.1. Study Selection and Characteristics

**Figure 2:**
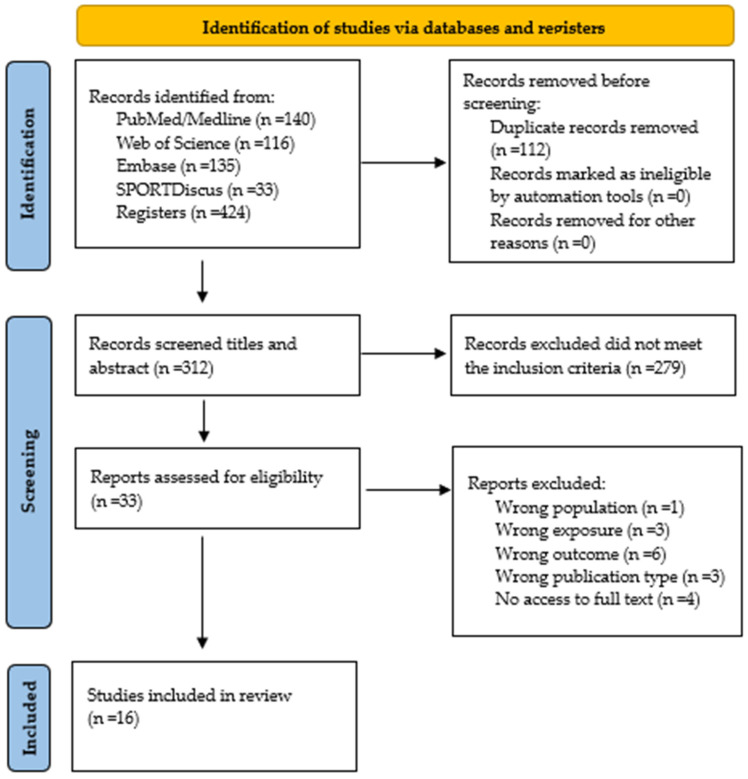
PRISMA 2020 flow diagram illustrating the study selection process.

Initially, a total of 424 studies were identified through searches in the databases (PubMed/Medline, Embase, SPORTDiscus, and Web of Science) in May 2025. Subsequently, 112 duplicate records were removed, leaving 312 articles for title and abstract screening. At this stage, 279 studies were excluded for not meeting the inclusion criteria. As a result, 34 articles were considered for full-text review, of which 16 met the eligibility criteria and were included in this systematic review (Figure 2). The studies excluded at this final stage are listed with their respective reasons for exclusion in Supplementary Table S3.

The characteristics of the included studies are summarized in Table 2. A total of 16 studies were included: 8 (50.0%) were prospective [27,34,35,36,37,38,39,40], 5 (31.3%) were cross-sectional [28,30,38,41,42], and 3 (18.7%) were case reports [31,32,33], published between 2012 and 2025. Of these, 4 studies (25.0%) were conducted in France [29,37,39,40], 3 (18.8%) in the United States [30,31,38], 2 studies each (12.5%) in Italy [27,33], Australia [32,35] and Brazil [28,41]; and 1 study each (6.3%) in England [36], Switzerland [42], and Finland [34].

Data were collected from 1389 athletes, of whom 874 (62.9%) were male, 146 (10.5%) were female, and 369 (26.6%) did not report participants’ sex [29,42]; ages ranged from 17 to 60 years. In terms of ultra-endurance modalities, 10 studies (62.5%) investigated ultramarathon events [27,29,30,32,33,36,37,39,40,41], 3 (18.8%) focused on triathlon [28,31,38], 2 (12.5%) on cycling [35,42], and 1 (6.2%) examined cross-country skiing [34].

**Table 2:** Summary of included studies that analyzed the association between sleep characteristics and physical or cognitive performance in ultra-endurance athletes.

Reference/Study Location	Study Design/Modality (Distance)/Collation Time	Sample Characteristics (*n*/Sex/Age)	Sleep Variables/Assessment Method	Performance Variables/Assessment Method	Main Results
***Physical Performance***					
Fitton et al. [35] Australia	Prospective Cycling-Tour de France Collect: 6 weeks (August to October)	N = 8 (M = 8/F = 0) Age: 30 (SD 4) y	TST, sleep onset time, and final awake time Method: Garmin wristwatch	PI, CTL, ATL, TSB; TSS Method: Power meters integrated into bicycle cranks (Watts)	↑ TST ↔ ↓ ATL and ↓ CTL ↓ TST; ↓ wake time and ↓ SQ ↔ ↑ PI
Mishica et al. [34] Finlandia	Prospective Cross-country skiing Collect: 16 weeks (August to November)	N = 29 (M = 15/F = 14) Age: 17 (SD 1) y	TST, SL, Method: Portable bedside ballistocardiographic sensor ^1^	CMJ and SRT Method: CMJ-force plataform ^2^ and TSR-treadmill ergometer ^3^	↑ TST ↔ ↓ blood lactate in SRT ↑ SL ↔ ↓ CMJ (Trend)
Daniel et al. [41] Brazil	Cross-sectional Ultramarathon-217 km Collect: Day before the competition	N = 38 (M = 32/F = 6) Age (M): 45 (SD 9.3) y (F): 44.8 (SD 5.1) y	TST, SL, SQ, SE, SDIS; use of medications and dysfunctions during the day Method: PSQI	Speed, classification, race time Method: Official race reports-Finishing position and TRT	Good QS: trained 1X more per week ↑ SL: Finalists TRT no association with SQ AS did not differ between good SQ and bad SQ
Anderson et al. [31] United States	Case study Triathlon (3.86 km swim, 180 km bike and 42.2 km run) Collect: Over 100 days	N = 1 (M = 1/F = 0) Age: 44 y	TST; SE; TLS; TDS; time awake; number of awakenings and sleep score Method: photoplethysmographic sensor (PPG) a Biostrap EVO	Swimming speed (m/s), cycling power (watts) and running speed (km/h). Method: Garmin Forerunner 945 GPS wristwatch	↑ TLS ↔ ↑ performance in swimming, cycling and running, being especially strong in cycling
Bianchi et al. [32] Australia	Case study Ultramarathon-326 km Collect: 7 days pre and 7 days post-race	N = 4 (M = 2/F = 2) Age: 45.5 (SD 3.1) y	TST, SL, SE; Bedtime; time to get up; time in bed; number of awakenings and subjective SQ Method: Wrist-worn activity monitor ^4^ and sleep diary	Race time Method: Runners’ delta time (expected time to finish − official time to finish)	Faster runners slept less than slower runners during the race (1.8 h vs. 9.0 h)
Kisiolek et al. [38] United States	Prospective Triathlon (10 km swim, 420.2 km bike, 84.4 km run) Collect: 2 days pre-race; after the pre-meeting and after stages 1 and 2 of the race = 4 nights of sleep	N = 17 (M = 14/F = 3) Age: 37.4 (SD 7.97) y	TST, SL, SE and waking episodes Method: Actigraphy ^5^	Race time Method: Time in each stage of the Triathlon	↑ TST ↔ ↓ performance on stage 1 and stage 3 ↑ SE ↔ ↑ slower performance on stage 2
Sinisgalli et al. [28] Brazil	Cross-sectional Triathlon (3.8 km swim, 180 km bike, 42.195 km run) Collect: 28~30 days before the race	N = 99 (M = 80/F = 19) Age (M): 39.0 (SD 5.7) y (F): 36.5 (SD 6.5) y	TST Method: Questionnaire question: Sleep time per night in the last week-Self-report	Race time Method: TRT	Performance those who sleep 4–6 h = those who sleep 7–8 h per night
Biorci et al. [33] Italy	Case study Ultramarathon-866 km-Transpyrenea race Collect: During the race-13 days of testing	N = 1 (M = 1/F = 0) Age: 48 y	TST Method: Self-report	Race time and Speed Method: AS (km/h	↑ TST ↔ ↑ AS (Each additional hour of sleep a 0.5 km/h increase in AS)
Martin et al. [30] United States	Cross-sectional Ultramarathon- ≤ 36 h, 36–60 h e >60 h Collect: Not reported	N = 636 (M = 541/F = 95) Age: 18–29 = 10.4% 30–39 = 31.1% 40–49 = 38,4% 50–59 = 16.4% >60 = 3.8%	TST, Sleep habits Method: Self-report	Race time Method: Self-reported (h)	TST and finish time showed ↔ positive for races lasting 36 to 60 h and >60 h
Poussel et al. [29] France	Cross-sectional Ultramarathon-168 km -Chamonix Collect: Post-race	N = 303 (sex not reported) Age: 44 (SD 7.5) y	Pre-race sleep management strategies (naps, increased sleep time, sleep deprivation); sleepiness during the race, drowsiness or other issues Method: Self-reported-post-race	Race time and classification Method: Recorded by the organizing team and (Delta-Time = Race Time–Target Time)	Athletes who did not sleep finished faster; ↑Race time: ↑ sleepiness during the race; ↑ TST the night before the race: completed the event faster ↑Time delta was: ↑ among those who slept
Knechtle et al. [42] Switzerland	Cross-sectional Cycling-Swiss Cycling Marathon-600 km Collect: Post-race	Finalists: N = 53 (sex not reported) Age: 46.0 (40.0–50.0) Non-finalists: N = 13 (sex not reported) Age: 45.0 (40.7–50.0) y	Nap-duration Method: Self-reported	Race time Method: Total time-finalists vs. non-finalists	Athletes who did not sleep completed the race faster; ↑ TST during the test ↔ ↑ TRT
***Cognitive Performance***					
Benchetrit et al. [36] England	Prospective Ultramarathon Collect: Sleep-During the 7 days prior to the race. Cognitive-on race day and post-race	N = 15 (M = 14/F = 1) Age: 40 (SD 8.6) y	TST, SQ and total time in bed Method: Loughborough daily sleep diary	Executive function; reaction time; decision-making Method: ANAM Battery (version 4); GNS; 2CRT, Stroop and Tower Puzzle	↑ SQ ↔ ↓ 2-choice reaction time and ↑ transfer rate in 2CRT
Bourlois et al. [37] France	Prospective Ultramarathon-168 km–Chamonix Collect: Supply point; end of nap and 1 km after the supply point	N = 23 (M = 22/F = 1) Age: Not reported	Strategy-nap Method: Duration of nap (set at 15 to 20 min) counting the time to fall asleep	Reaction time Method: BlazePod^®^	Napping during: faster reaction times. After napping, reaction times improved up to 1 km after the checkpoint.
Tonacci et al. [27] Italy	Prospective Ultramarathon-866 km-Transpyrenea race Collect:4 sessions (one pre, two during-km 166 and km 418 and one post-race)	N = 40 (M = 36/F = 4) Age: 43.0 (SD 8.8) y	TST and TST normalized Method: Structured questionnaire	Language control and executive function Method: Language: (COWAT abbreviated ^6^); TMT-A; TMT-B, TMT	TST no association cognitive variables.
***Physical And Cognitive Performance***					
Hurdiel et al. [39] France	Prospective Ultramarathon-168 km-Chamonix Collect: Pre-race (in the 24 h before) and during the race (8 assessments along the checkpoint)	N = 92 (M = 92/F = 0) Age: 43 (SD 7.52) y	Time in bed the night before the race and TST on the course Method: Self-report	Physical: Race time Cognitive: number of correct answers Method: Digit Symbol Substitution Task ^7^	↑ TST during the test ↔ ↑ TRT ↑ Greater accumulated TST before testing ↔ ↓ DSST performance (+1 h of sleep: less 2.7 correct responses on the DSST)
Hurdiel et al. [40] France	Prospective Ultramarathon-168 km-Chamonix Collect: Pre-race and post-race	N = 17 (M = 16/F = 1) Age: 43.4 (SD 6.4) y	TST Method: Triaxial actigraph ^8^ and self-reported (rest during the test)	Physical: Race time Cognitive: psychomotor vigilance Method: Simple 10-min serial response time tests ^9^-pre- and post-run	↑ TST during the test ↔ ↑ TRT TST no association cognitive performance

Legend: ↑ greater values; ↓ lower values; ↔ indicates a statistical association between variables; = no significant difference; y: years; SD: standard deviation; F: female; M: male; km: kilometers; h: hour; min: minutes; m: meters; s: second; DSST: Digit symbol substitution task; COWAT: Controlled oral word association test; GNS: general neuropsychological screening; 2CRT: 2-choice reaction time; TST: Total Sleep Time; SL: sleep latency; SQ: sleep quality; SE: sleep efficiency; SDIS: sleep disorders; PSQI: Pittsburgh sleep quality index; TLS: time in light sleep; TDS: time in deep sleep; CMJ: maximal countermovement jumps; SRT: submaximal running tests; PI: performance index; CTL: chronic training load; ATL: acute training load; TSB: training stress balance; TSS: training stress score; TMT-A: Cognitive processing speed; TMT-B: attention and sequencing skills; TMT: Computerized version of the Trail Making Test–pre- and post-assessment; AS: average speed; TRT: Total race time; ^1^—(EMFIT QS, Emfit OY, Jyväskylä, Finland); ^2^—CMJ (Force platform (HUR FP8, HUR Oy, Kokkola, Finland); ^3^—TSR (Tunturi GO Run 50 treadmill-Tunturi Fitness, Flevoland, The Netherlands); ^4^—(Actical, Minimitter, Philips Respironics, Bend, OR, USA); ^5^—(Fatigue Science, Vancouver, BC, Canada); ^6^—version; 1989; ^7^—(DSST; Wechsler, 1981); ^8^—(GT3X, TheActiGraph, Pensacola, FL, USA); ^9^—(Wilkinson & Houghton, 1982).

### 3.2. Sleep Assessment in Ultra-Endurance Athletes–Parameters, Methods, and Timing

Most of the included studies examined sleep exposure through more than one marker in ultra-endurance athletes. The most frequently sleep marker was total sleep time (TST), reported in 13 studies (81.3%) [27,28,30,31,32,33,34,35,36,38,39,40,41], followed by sleep latency (SL) [32,34,38,41] and sleep efficiency (SE) [31,32,38,41], each reported in four studies (25.0%), while SQ was assessed in three studies (18.8%) [32,36,41]. In addition to core sleep parameters, several studies also addressed pre-race sleep management strategies, such as napping, sleep extension, intentional sleep deprivation [29], general sleep habits [30], and nap timing and duration [42].

Regarding the tools and approaches used to assess sleep, objective methods included actigraphy [32,38,40], while subjective methods involved validated questionnaires such as the Pittsburgh Sleep Quality Index (PSQI) [41], sleep diaries [32,36], photoplethysmographic (PPG) sensors [31], ballistocardiography [34], and wrist-worn monitors such as Garmin devices [35]. In the vast majority of cases, sleep was self-reported through structured questions developed by the authors of each study [27,28,29,30,33,37,39,42].

The timing of data collection varied across studies. Some focused on a single time point, such as the pre-race period [28,34,36,41], others monitored sleep during the competition [31,33,35,37], and some examined post-race sleep recovery [29,42]. Additionally, certain studies aimed to compare sleep across different time points, including pre- vs. post-race [32,38,40], pre- vs. during-race [39], and even pre- vs. during- vs. post-race periods [27].

### 3.3. Association Between Sleep Characteristics and Physical Performance

Regarding to performance outcomes, 11 studies (68.8%) analyzed the association between sleep characteristics and physical performance in ultra-endurance athletes [28,29,30,31,32,33,34,35,38,41].

Among the studies conducted with cycling athletes, the main findings indicated that longer TST was associated with lower acute and chronic training loads. Conversely, higher performance indices were negatively correlated with TST, wake time, and SQ [35]. Additionally, athletes who did not sleep during the race finished faster; therefore, a longer TST was associated with longer race times [42]. Similar findings were observed among ultramarathon runners, although race time was positively correlated with sleepiness during the competition, and extended sleep on the night prior to the race was associated with improved performance [29].

Still regarding ultramarathon athletes, greater SL was observed among the finalists of a 217 km race, and athletes with better SQ reported training one additional day per week; however, no association was found between sleep markers and race time [41]. In a 326 km ultramarathon, athletes who slept less during the race showed better performance [32]. In an 866 km event, each additional hour of TST was associated with a 0.5 km/h increase in average speed [33]. TST was positively associated with finish time in races lasting between 36 to 60 h and over 60 h [30].

Among skiers, each additional hour of TST was associated with a 0.62 mmol/L reduction in blood lactate concentration during submaximal running, and there was a trend toward greater SL being associated with lower countermovement jump performance [34].

Finally, among triathletes, one study conducted over a 100-day period reported a positive association between increased time in light sleep (TLS) was positively associated with performance across all three modalities, with the strongest effect observed in cycling [31]. In another study, a higher TST was associated with faster performance in stages 1 and 3, while higher sleep efficiency (SE) was associated with slower performance in stage 2 [38]. Additionally, A previous cross-sectional study found no difference in race time between triathletes who slept 4–6 h and those who slept 7–8 h per night [28].

### 3.4. Sleep and Cognitive Performance

Although most studies focused on physical performance, three studies (18.8%) [27,36,37] evaluated the impact of sleep on cognitive performance in ultramarathon athletes. In one study, pre-competition SQ showed a moderate correlation with reaction time and transfer rate on the two-choice reaction time test (2CRT) [36]. In a 168-km race that assessed the effects of napping during competition, a significant improvement in reaction time was observed immediately after the nap [37]. No association was found between TST and cognitive performance variables [27].

### 3.5. Simultaneous Association Between Sleep and Physical and Cognitive Performance

Two studies (12.5%) [39,40] simultaneously examined the association between sleep parameters and both physical and cognitive performance.

During the 168 km ultramarathon in Chamonix (2018), race time was positively correlated with sleep during the event. Greater accumulated sleep before cognitive testing was linked to poorer performance on the Digit Symbol Substitution Task (DSST), with each additional hour of sleep corresponding to 2.7 fewer correct answers [39]. In a previous study from the same authors (2015 edition), a similar association was found between sleep and race time, but no correlation was observed between sleep and cognitive performance [40].

## 4. Discussion

This systematic review examined the available evidence on the association between sleep parameters and the physical and cognitive performance of ultra-endurance athletes. Among the findings, both sleep quantity and quality parameters were investigated, with evidence supporting their relevance to physical performance. However, this relationship appeared to be potentially non-linear, influenced by the competitive context and individual sleep strategies. Regarding cognitive performance, strategies such as pre-competition sleep extension and in-race napping demonstrated benefits, particularly in functions related to attention and reaction time.

Regarding physical performance, the results were heterogeneous, with most studies identifying significant associations between sleep variables and performance indicators. While greater TST was associated with better performance when observed pre-race, particularly in the context of sleep extension strategies [29], higher TST during competition was linked to slower race completion times [39,40,42]. These findings highlight that total sleep time should not be considered in isolation, as similar sleep duration may be associated with different levels of well-being and perceived workload. Sleep quality, recovery status, and underlying physiological mechanisms influence physical functioning and cognitive performance, thereby shaping how sleep relates to performance outcomes [14,15]. Furthermore, beyond training and competition demands, lifestyle-related factors may further impair sleep quality in athletes [10]. Evening exposure to blue light from electronic devices [43] and inadequate hydration [44] have been associated with circadian disruption, poorer sleep quality, and increased cognitive fatigue. Although not consistently assessed in the included studies, these factors may contribute to sleep disturbances in endurance athletes.

In extreme competitive contexts, this pattern suggests that sleep may be suppressed in favor of performance, and that athletes with greater fitness or higher physical and mental resilience may better tolerate acute sleep restriction [45]. Moreover, sleep extension adopted prior to competition appears to act as a potential protective factor for performance. Thus, pre-competition sleep extension has emerged as a viable strategy to minimize the effects of sleep debt and has shown benefits for athletes’ physical performance [46,47].

Conversely, longer sleep duration during competition may serve as a marker of accumulated fatigue or reduced capacity to tolerate exertion, which could explain its association with poorer performance. This may indicate that, in competitive contexts, elite athletes tend to prioritize performance over sleep or may exhibit greater resilience to short-term sleep restriction. However, chronic sleep reduction can lead to long-term health impairments [48,49], including mood disorders, increased risk of accidents, and the development of metabolic and cardiovascular conditions such as diabetes, hypertension, and heart disease [50].

Some studies have shown the effect of sleep on performance in different disciplines, allowing us to better understand this relationship. Among cyclists, greater TST was correlated with reductions in both acute and chronic training loads, whereas higher performance indices were associated with shorter TST, shorter wake time, and poorer SQ [35]. In skiers, longer sleep duration was associated with lower blood lactate concentrations during submaximal running, while increased SL was linked to poorer performance in the countermovement jump [34]. Among triathletes, longer time spent in light sleep was associated with better performance across all three modalities [31]. Additionally, greater TST was associated with shorter completion times in stages 1 and 3 of a three-day triathlon. Interestingly, higher sleep efficiency was linked to poorer performance in stage 2 [38]. A cross-sectional study found no significant difference in performance between triathletes who slept 4–6 h and those who slept 7–8 h per night [28].

Moreover, although adequate sleep is essential for physiological and psychological well-being [10,11,12,51] and is widely recognized as a vital component of athletic training and post-competition recovery [51], most athletes tend to experience poor sleep in both quality and quantity. Despite the general recommendation for adults to obtain between 7 and 9 h of sleep per night [18], athletes often fall short of this target, commonly reporting difficulties in sleep initiation and maintenance [10,36]. These issues may, in turn, help explain our findings regarding the influence of sleep exposure on physical performance outcomes.

Regarding cognitive performance, better pre-race SQ was associated with higher performance outcomes on reaction time and transfer rate tests (2CRT) [36], suggesting a protective effect of adequate sleep against cognitive impairments induced by ultra-endurance participation. Naps taken during competition led to significant improvements in reaction time [37], whereas greater accumulated TST prior to cognitive testing was associated with poorer performance on the Digit Symbol Substitution Task, with a reduction of 2.7 correct responses for each additional hour of sleep [39].

The findings of the present review reinforce that the neurocognitive performance deteriorates with sleep restriction and does not fully recover even after more than a week of rebound sleep [52]. Furthermore, longer ultra-endurance events are accompanied by more extreme physiological responses, resulting in performance declines at various points during the event [53], especially when compounded by sleep deprivation. Weekend sleep recovery is insufficient to fully restore sleep debt or reverse neurobehavioral impairments in adults subjected to sleep restriction [54]. Therefore, both TST and sleep efficiency play a crucial role in supporting adequate recovery following ultra-endurance events.

Another important factor identified in our review for enhancing cognitive performance is the implementation of napping during competitions [37]. To support this finding, a previous systematic review investigating the impact of sleep interventions on athletic performance highlighted a positive effect of napping on cognitive outcomes, identifying it as the most impactful sleep intervention [47]. Indeed, naps and brief episodes of sleep may serve as complementary recovery strategies when consolidated and prolonged sleep is not feasible [54]. Furthermore, chronotype may influence the effectiveness of sleep strategies, as circadian preference affects the sleep–wake cycle and alertness [55]. Therefore, standardized sleep recommendations may be insufficient, and individualized chronotype-based strategies may be more appropriate for ultra-endurance athletes.

The findings of this review reinforce the importance of incorporating sleep strategies into the training and competition planning of ultra-endurance athletes, with a focus on promoting pre-competition sleep extension, scheduled naps, and regular monitoring of SQ and quantity throughout preparation and post-race recovery. In this context, it is essential that athletes and their coaches recognize the critical role of effective sleep management in optimizing athletic performance.

Moreover, many of the included studies assessed sleep using subjective methods, often relying on self-reported measures [27,28,29,33,37,39,40,42], what may explain the conflicting findings. While such measures provide valuable insights into athletes’ perceptions, combining them with objective tools, such as actigraphy, could enhance the accuracy of future investigations [45]. Sleep regularity, assessed through actigraphy metrics, reflects the consistency of the sleep–wake cycle on a day-to-day basis and has been associated with physiological recovery and cognitive functioning [56,57,58]. Similarly, social jet lag, typically assessed by the Munich Chronotype Questionnaire (MCTQ), captures the misalignment between biological and social sleep schedules and has been associated with adverse health and performance outcomes [59,60].

This review presents several strengths, including methodological rigor and a comprehensive literature search. To the best of our knowledge, it is the first systematic review to comprehensively synthesize the evidence on the association between sleep parameters and both physical and cognitive performance in ultra-endurance athletes. However, some limitations should be acknowledged. There was considerable methodological heterogeneity among the included studies, with notable variations in study design (cross-sectional, prospective, and case studies). Moreover, few studies adequately controlled for important confounding variables such as age, sex, training level, and environmental conditions during competition, which may have influenced the observed associations. Additionally, although inclusion criteria were based on participation in ultra-endurance events, many included studies did not stratify outcomes by sex or sport. Given known differences in physiological demands and daily circadian rhythms across disciplines and between male and female athletes, this lack of stratification may have limited the interpretation of sport-specific and sex-specific associations between sleep parameters and performance. Finally, many studies relied on self-reported sleep measures rather than objective tools such as actigraphy or polysomnography, potentially reducing measurement validity and introducing recall or cultural bias.

These limitations highlight the need for cautious interpretation of the findings and underscore the need for future research to employ more robust study designs, ensure rigorous control of confounding factors, and standardization in the measurement of sleep and performance variables, and conduct sex- and sport-specific analyses in ultra-endurance athletes.

## 5. Conclusions

In this systematic review, most studies identified associations between sleep parameters, particularly TST, and the physical and/or cognitive performance of ultra-endurance athletes. Strategies such as pre-race sleep extension and napping during competition directly influenced race performance. Although less investigated, the influence of sleep on cognitive performance suggests potential benefits, particularly in maintaining attention and reaction time during prolonged events. It should be noted, however, that the available evidence predominantly reflects data from male athletes, due to the limited representation of female participants in the included studies, which may restrict the generalizability of these findings. In this context, sleep should be considered a relevant factor in the planning and monitoring of ultra-endurance athletes, with the goal of optimizing both performance and safety in extreme endurance settings, while also considering sex-related differences. Finally, future research is needed to further elucidate the underlying mechanisms and to support the development of evidence-based interventions.

## Figures and Tables

**Table 1:** PECOS criteria adopted in this systematic literature review.

PECOS	Inclusion Criteria
**P**articipants	Ultra-endurance athletes of both sexes (humans)
	Type of ultra-endurance sport considered: running, cycling, swimming, triathlon, rowing, skiing, or adventure racing.
**E**xposition	Sleep efficiency, sleep duration, sleep habits, sleep strategies, sleep disorders, and sleep quality
**C**omparative or control	Lower and higher sleep quality/quantity; short and long sleep duration
**O**utcome measurement	Physical or Cognitive performance in ultra-endurance events.
	*Objective physical performance outcomes include:*
	Time to complete;
	Final ranking or placement;
	Maximal oxygen uptake (VO_2_max);
	Muscular power;
	Time to exhaustion;
	Power output, average speed, and distance covered;
	Countermovement Jump Performance
	Reduction in performance compared to baseline.
	*Cognitive performance outcomes may include:*
	Reaction time;
	Decision-making ability;
	Mental confusion;
	Executive functions (e.g., working memory and cognitive flexibility);
	Attention;
	Psychomotor performance.
**S**tudies included	Clinical trials, observational studies (cross-sectional, cohort), or case–control studies; relate case

## Data Availability

No new data were created or analyzed in this study. Data sharing is not applicable to this article.

## Supplementary Materials

The following supporting information can be downloaded at: https://www.mdpi.com/article/10.3390/jcm15041398/s1, Table S1 PRISMA 2020 Checklist; Table S2 Complete literature Search; Table S3 Deleted articles.
