# Supplementary material for: The Role of Sleep on Physical and Cognitive Performance of Ultra-Endurance Athletes: A Systematic Review
Source: J Clin Med. 2026 Feb 10;15(4):1398. doi: 10.3390/jcm15041398 (PMC12941826; doi:10.3390/jcm15041398)
Supplement: Supplementary file 1 [file jcm-15-01398-s001.zip › jcm-4105962-supplementary.pdf]

The role of sleep on physical and cognitive performance of ultra-endurance athletes: a systematic review

Larissa Quintão Guilherme[1], Bruno Otávio Rodrigues[2], Carla de Oliveira Barbosa Rosa[2], Luciano Bernardes Leite[1], Pedro Forte[4], Volker Scheer[5], Helen Hermana Miranda Hermsdorff[3], Ana Claudia Pelissari Kravchychyn[3], Helton de Sá Souza[1]

[1] Department of Physical Education, Federal University of Viçosa (UFV), Viçosa, MG, Brazil; larissa.guilherme@ufv.br (L.Q.G.); luciano.leite@ufv.br (L.B.L.); helton.souza@ufv.br (H.d.S.S.)

[2] Laboratory of Food Intake, Department of Nutrition and Health, Federal University of Viçosa (UFV), Viçosa, MG, Brazil; bruno.o.rodrigues@ufv.br (B.O.R); carla.rosa@ufv.br (C.d.O.B.R.)

[3] Laboratory of Clinical Analysis and Genomics; Laboratory of Energy Metabolism and Body Composition, Department of Nutrition and Health, Federal University of Viçosa (UFV), Viçosa, MG, Brazil; ana.pelissari@ufv.br (A.C.P.K.); helenhermana@ufv.br (H.H.M.H.)

[4] Department of Sports Sciences, Instituto Politécnico de Bragança, Bragança, Portugal; pedromiguelforte@gmail.com (P.F.)

[5] Ultra Sports Science Foundation, 109 Boulevard de l’Europe, 69310, Pierre-Benite, France; volkerscheer@yahoo.com (V.S.)

Supplementary Table S1. PRISMA 2020 Checklist

Section and Topic	Item #	Checklist item	Location where item is reported
**TITLE**			
Title	1	Identify the report as a systematic review.	#1
**ABSTRACT**			
Abstract	2	See the PRISMA 2020 for Abstracts checklist.	#2
**INTRODUCTION**			
Rationale	3	Describe the rationale for the review in the context of existing knowledge.	#3 and 4
Objectives	4	Provide an explicit statement of the objective(s) or question(s) the review addresses.	#4
**METHODS**			
Eligibility criteria	5	Specify the inclusion and exclusion criteria for the review and how studies were grouped for the syntheses.	#4 and 5
Information sources	6	Specify all databases, registers, websites, organisations, reference lists and other sources searched or consulted to identify studies. Specify the date when each source was last searched or consulted.	#6
Search strategy	7	Present the full search strategies for all databases, registers and websites, including any filters and limits used.	#6 and 7
Selection process	8	Specify the methods used to decide whether a study met the inclusion criteria of the review, including how many reviewers screened each record and each report retrieved, whether they worked independently, and if applicable, details of automation tools used in the process.	#7 and 8
Data collection process	9	Specify the methods used to collect data from reports, including how many reviewers collected data from each report, whether they worked independently, any processes for obtaining or confirming data from study investigators, and if applicable, details of automation tools used in the process.	#7 and 8
Data items	10a	List and define all outcomes for which data were sought. Specify whether all results that were compatible with each outcome domain in each study were sought (e.g. for all measures, time points, analyses), and if not, the methods used to decide which	#5

Section and Topic	Item #	Checklist item	Location where item is reported
		results to collect.	
	10b	List and define all other variables for which data were sought (e.g. participant and intervention characteristics, funding sources). Describe any assumptions made about any missing or unclear information.	#5
Study risk of bias assessment	11	Specify the methods used to assess risk of bias in the included studies, including details of the tool(s) used, how many reviewers assessed each study and whether they worked independently, and if applicable, details of automation tools used in the process.	#8
Effect measures	12	Specify for each outcome the effect measure(s) (e.g. risk ratio, mean difference) used in the synthesis or presentation of results.	#8
Synthesis methods	13a	Describe the processes used to decide which studies were eligible for each synthesis (e.g. tabulating the study intervention characteristics and comparing against the planned groups for each synthesis (item #5)).	#8 and 9
	13b	Describe any methods required to prepare the data for presentation or synthesis, such as handling of missing summary statistics, or data conversions.	
	13c	Describe any methods used to tabulate or visually display results of individual studies and syntheses.	#8 and 9
	13d	Describe any methods used to synthesize results and provide a rationale for the choice(s). If meta-analysis was performed, describe the model(s), method(s) to identify the presence and extent of statistical heterogeneity, and software package(s) used.	
	13e	Describe any methods used to explore possible causes of heterogeneity among study results (e.g. subgroup analysis, meta-regression).	
	13f	Describe any sensitivity analyses conducted to assess robustness of the synthesized results.	
Reporting bias assessment	14	Describe any methods used to assess risk of bias due to missing results in a synthesis (arising from reporting biases).	#8
Certainty assessment	15	Describe any methods used to assess certainty (or confidence) in the body of evidence for an outcome.	#8 and 9
**RESULTS**			
Study selection	16a	Describe the results of the search and selection process, from the number of records identified in the search to the number of studies included in the review, ideally using a flow diagram.	#8 and 9

Section and Topic	Item #	Checklist item	Location where item is reported
	16b	Cite studies that might appear to meet the inclusion criteria, but which were excluded, and explain why they were excluded.	#8 and 9
Study characteristics	17	Cite each included study and present its characteristics.	#9 - 15
Risk of bias in studies	18	Present assessments of risk of bias for each included study.	#18 - 21
Results of individual studies	19	For all outcomes, present, for each study: (a) summary statistics for each group (where appropriate) and (b) an effect estimate and its precision (e.g. confidence/credible interval), ideally using structured tables or plots.	#11 - 15
Results of syntheses	20a	For each synthesis, briefly summarise the characteristics and risk of bias among contributing studies.	#18 - 21
	20b	Present results of all statistical syntheses conducted. If meta-analysis was done, present for each the summary estimate and its precision (e.g. confidence/credible interval) and measures of statistical heterogeneity. If comparing groups, describe the direction of the effect.	
	20c	Present results of all investigations of possible causes of heterogeneity among study results.	
	20d	Present results of all sensitivity analyses conducted to assess the robustness of the synthesized results.	
Reporting biases	21	Present assessments of risk of bias due to missing results (arising from reporting biases) for each synthesis assessed.	#18 - 19
Certainty of evidence	22	Present assessments of certainty (or confidence) in the body of evidence for each outcome assessed.	
**DISCUSSION**			
Discussion	23a	Provide a general interpretation of the results in the context of other evidence.	#21 – 24
	23b	Discuss any limitations of the evidence included in the review.	#24
	23c	Discuss any limitations of the review processes used.	#24
	23d	Discuss implications of the results for practice, policy, and future research.	#24

Section and Topic	Item #	Checklist item	Location where item is reported
**OTHER INFORMATION**			
Registration and protocol	24a	Provide registration information for the review, including register name and registration number, or state that the review was not registered.	#4
	24b	Indicate where the review protocol can be accessed, or state that a protocol was not prepared.	#4
	24c	Describe and explain any amendments to information provided at registration or in the protocol.	
Support	25	Describe sources of financial or non-financial support for the review, and the role of the funders or sponsors in the review.	#25
Competing interests	26	Declare any competing interests of review authors.	#25
Availability of data, code and other materials	27	Report which of the following are publicly available and where they can be found: template data collection forms; data extracted from included studies; data used for all analyses; analytic code; any other materials used in the review.	#25

From: Page MJ, McKenzie JE, Bossuyt PM, Boutron I, Hoffmann TC, Mulrow CD, et al. The PRISMA 2020 statement: an updated guideline for reporting systematic reviews. BMJ 2021;372:n71. doi: 10.1136/bmj.n71. This work is licensed under CC BY 4.0. To view a copy of this license, visit https://creativecommons.org/licenses/by/4.0/

Supplementary Table S2. Complete literature search

Information Source		Search Strategy	Items foun
EMBASE	#1	(‘ultra-endurance running’ OR ‘ultra marathon’ OR ‘ultramarathon running’ OR ‘ultra-endurance’ OR ‘ultra-athlete’ OR ‘ultra-endurance training’ OR ‘ultra-distance’ OR **‘ultramarathon’/exp** OR ‘ultra-event’ OR ‘trail run’ OR ‘sky run’ OR ‘fell run’ OR ‘off-road run’ OR ‘mountain run’ OR ‘ultra run’ OR ‘ultra trail’ OR ‘ultra endurance’ OR ‘**Skiing’/exp** OR ‘cross country skiing’ OR ‘cross country ski’ OR ‘cross country skiers’ OR skating OR ‘ironman’ OR ‘triathl’ OR ‘Bicycling’ OR ‘ultraendurance sports’ OR **‘treadmill exercise’/exp** OR ‘treadmill exercise’ OR **‘high intensity exercise’/exp** OR ‘high intensity exercise’) AND [embase]/lim	56.285
	#2	(‘**sleep’/exp** OR ‘sleep’ OR ‘sleeping habits’ OR ‘sleep habits’ OR ‘sleep duration’ OR **‘sleep hygiene’/exp** OR ‘sleep hygiene’ OR **‘sleep quality’/exp** OR ‘sleep quality’ OR ‘**sleep latency’/exp** OR ‘sleep latency’ OR **‘sleep stage’/exp** OR ‘sleep stage’ OR ‘sleepiness’ OR ‘sleep problem’ OR ‘**sleep disorder’/exp** OR ‘sleep disorder’ OR ‘sleep wake disorders’ OR **‘sleep deprivation’/exp** OR ‘sleep deprivation’ ‘insufficient sleep’ OR ‘sleep fragmentation’ OR ‘**fragmented sleep’/exp** OR ‘fragmented sleep’ OR ‘sleep loss’ OR ‘restricted sleep’ OR ‘sleep restriction’ OR ‘**sleep time’/exp** OR ‘sleep time’ OR ‘Night sleep’ OR **‘Night sleep’/exp** OR ‘sleep pattern’ OR **‘sleep pattern’/exp** OR ‘sleep spindle’ OR **‘sleep spindle’/exp** OR ‘sleep stage’ OR **‘sleep stage’/exp** OR ‘somnolence’ OR **‘somnolence’/exp** OR ‘sleep debt’ OR **‘sleep debt’/exp)** AND [embase]/lim	192.383
	#3	('physical functional performance' OR **'athletic performance'/exp** OR 'athletic performance' OR **'physical performance'/exp** OR 'physical performance’ OR **'psychomotor performance'/exp** OR 'psychomotor performance’ OR **‘mental performance’/exp** OR ‘mental performance’ OR 'cognitive performance' OR 'test time' OR 'final ranking' OR 'placement' OR **'maximal oxygen uptake'/exp** OR **‘**maximal oxygen uptake’ OR 'vo2max' OR 'muscular power' OR 'time to exhaustion' OR 'power output' OR 'average speed' OR 'distance covered' OR **'reaction time'/exp** OR	2.390.231

		'reaction time' OR 'response speed' OR 'response time' OR 'response times' OR **'decision making'/exp** OR 'decision making' OR **'executive function'/exp** OR 'executive function' OR 'executive functions' OR **'cognitive flexibility'/exp** OR 'cognitive flexibility' OR 'cognitive flexibilities' OR **'attention'/exp** OR 'attention' OR 'focus of attention' OR 'attention focus' OR 'decision-making ability' OR 'mental confusion' OR 'working memory') AND [embase]/lim	
		#1 AND #2 AND #3	135
**PUBMED**	#1	"ultra-endurance running" OR "ultra marathon" OR "ultramarathon running" OR "ultra-endurance" OR "ultra-athlete" OR "ultra-endurance training" OR "ultra-distance" OR "ultramarathon" OR "ultra-event" OR "trail run" OR "mountain run" OR "ultra run" OR "ultra trail" OR "ultra endurance" OR "cross country skiing" OR "cross country ski" OR "cross country skiers" OR "skating" OR "ironman" OR "triathlon" OR "bicycling"[MeSH Terms] OR "bicycling" OR "ultraendurance sports" OR "treadmill exercise" OR "high intensity exercise"	31.312
	#2	"sleep"[MeSH Terms] OR "sleep" OR "sleeping habits" OR "sleep habits" OR "sleep duration"[MeSH Terms] OR "sleep duration" OR "sleep hygiene"[MeSH Terms] OR "sleep hygiene" OR "sleep quality"[MeSH Terms] OR "sleep quality" OR "sleep latency"[MeSH Terms] OR "sleep latency" OR "sleep stage" OR "sleep stages"[MeSH Terms] OR "sleep stages" OR "sleepiness"[MeSH Terms] OR "sleepiness" OR "sleep problem" OR "sleep disorder" OR "sleep wake disorders"[MeSH Terms] OR "sleep wake disorders" OR "sleep deprivation"[MeSH Terms] OR "sleep deprivation" OR "insufficient sleep" OR "sleep fragmentation" OR "fragmented sleep" OR "sleep loss" OR "restricted sleep" OR "sleep restriction" OR "sleep time" OR "night sleep" OR "sleep pattern" OR "sleep patterns" OR "somnolence" OR "sleep debt" OR "sleep spindle" OR "sleep spindles"	324.787
	#3	"Physical Functional Performance"[MeSH Terms] OR "Physical Functional Performance" OR "Athletic Performance"[MeSH Terms] OR "Athletic Performance" OR "Physical performance" OR "Psychomotor Performance"[MeSH Terms] OR "Psychomotor Performance" OR "Cognitive performance" OR "test time" OR "Final ranking" OR "Placement" OR "Maximal oxygen uptake" OR "VO_2max" OR "Muscular power" OR "Time to exhaustion" OR "Power output" OR "Average speed" OR "distance covered" OR "Reaction	1.663.845

		Time"[MeSH Terms] OR "Reaction Time" OR "Response Speed" OR "Response Time" OR "Response Times" OR "Decision Making"[MeSH Terms] OR "Decision Making" OR "Executive Function"[MeSH Terms] OR "Executive Function" OR "Executive Functions" OR "Cognitive Flexibility"[MeSH Terms] OR "Cognitive Flexibility" OR "Cognitive Flexibilities" OR "Attention"[MeSH Terms] OR "Attention" OR "Focus of Attention" OR "Attention Focus" OR "Decision-making ability" OR "Mental confusion" OR "Working memory"	
		#1 AND #2 AND #3	140
**SPORTDiscus**	#1	"ultra-endurance running" OR "ultra marathon" OR "ultramarathon running" OR "ultra-endurance" OR "ultra-athlete" OR "ultra-endurance training" OR "ultra-distance" OR "ultramarathon" OR "ultra-event" OR "trail run" OR "mountain run" OR "ultra run" OR "ultra trail" OR "ultra endurance" OR "cross country skiing" OR "cross country ski" OR "cross country skiers" OR "skating" OR "ironman" OR "triathlon" OR "bicycling" OR "ultraendurance sports" OR "treadmill exercise" OR "high intensity exercise"	68.692
	#2	"sleep" OR "sleeping habits" OR "sleep habits" OR "sleep duration" OR "sleep hygiene" OR "sleep quality" OR "sleep latency" OR "sleep stage" OR "sleep stages" OR "sleepiness" OR "sleep problem" OR "sleep disorder" OR "sleep wake disorders" OR "sleep deprivation" OR "insufficient sleep" OR "sleep fragmentation" OR "fragmented sleep" OR "sleep loss" OR "restricted sleep" OR "sleep restriction" OR "sleep time" OR "night sleep" OR "sleep pattern" OR "sleep patterns" OR "somnolence" OR "sleep debt" OR "sleep spindle" OR "sleep spindles"	15.974
	#3	"Physical Functional Performance" OR "Athletic Performance" OR "Physical performance" OR "Psychomotor Performance" OR "Cognitive performance" OR "Time to complete the race" OR "Final ranking" OR "Placement" OR "Maximal oxygen uptake" OR "VO_2max" OR "Muscular power" OR "Time to exhaustion" OR "Power output" OR "Average speed" OR "Distance covered" OR "Reaction Time" OR "Response Speed" OR "Response Time" OR "Response Times" OR "Decision Making" OR "Executive Function" OR "Executive Functions" OR "Cognitive Flexibility" OR "Cognitive Flexibilities" OR "Attention" OR "Focus of Attention" OR "Attention Focus" OR	95.246

		“Decision-making ability” OR “Mental confusion” OR “Working memory”	
		#1 AND #2 AND #3	33
**Web of Science**	#1	ALL=("ultra-endurance running" OR "ultra marathon" OR "ultramarathon running" OR "ultra-endurance" OR "ultra-athlete" OR "ultra-endurance training" OR "ultra-distance" OR "ultramarathon" OR "ultra-event" OR "trail run" OR "mountain run" OR "ultra run" OR "ultra trail" OR "ultra endurance" OR "cross country skiing" OR "cross country ski" OR "cross country skiers" OR "skating" OR "ironman" OR "triathlon" OR "bicycling" OR "ultraendurance sports" OR “treadmill exercise” OR “high intensity exercise”)	27.131
	#2	ALL=("sleep" OR "sleeping habits" OR "sleep habits" OR "sleep duration" OR "sleep hygiene" OR "sleep quality" OR "sleep latency" OR "sleep stage" OR "sleep stages" OR "sleepiness" OR "sleep problem" OR "sleep disorder" OR "sleep wake disorders" OR "sleep deprivation" OR "insufficient sleep" OR "sleep fragmentation" OR "fragmented sleep" OR "sleep loss" OR "restricted sleep" OR "sleep restriction" OR "sleep time" OR "night sleep" OR "sleep pattern" OR "sleep patterns" OR "somnolence" OR "sleep debt" OR "sleep spindle" OR "sleep spindles")	425.909
	#3	ALL=(“Physical Functional Performance” OR “Athletic Performance” OR “Physical performance” OR “Psychomotor Performance” OR “Cognitive performance” OR “Time to complete the race” OR “Final ranking” OR “Placement” OR “Maximal oxygen uptake” OR “VO_2max” OR “Muscular power” OR “Time to exhaustion” OR “Power output” OR “Average speed” OR “Distance covered” OR “Reaction Time” OR “Response Speed” OR “Response Time” OR “Response Times” OR “Decision Making” OR “Executive Function” OR “Executive Functions” OR “Cognitive Flexibility” OR “Cognitive Flexibilities” OR “Attention” OR “Focus of Attention” OR “Attention Focus” OR “Decision-making ability” OR “Mental confusion” OR “Working memory”)	2.977.254
		#1 AND #2 AND #3	116

Supplementary Table S3. Deleted articles

Exclusion Criteria
**Wrong outcome (n=6)**
Brager AJ, Demiral S, Choynowski J, Kim J, Campbell B, Capaldi VF, Simonelli G, Hammer S. Earlier shift in race pacing can predict future performance during a single-effort ultramarathon under sleep deprivation. Sleep Sci. 2020 Jan-Mar;13(1):25-31. doi: 10.5935/1984-0063.20190132. PMID: 32670489; PMCID: PMC7347363.
Mann DL, Pattinson CL, Allan A, St Pierre L, Staton S, Thorpe K, Rossa K, Smith SS. Sleep deprivation and recovery: Endurance racing as a novel model. Eur J Sport Sci. 2024 Aug;24(8):1176-1185. doi: 10.1002/ejsc.12143. Epub 2024 Jun 14. PMID: 38874812; PMCID: PMC11295088.
Nédélec M, Chauvineau M, Guilhem G. On the Road to Camarón: The Sleep of an Ultra-Endurance Athlete Cycling 10,000 km in 24 Days. Int J Environ Res Public Health. 2022 Apr 9;19(8):4543. doi: 10.3390/ijerph19084543. PMID: 35457410; PMCID: PMC9025025.
Netzer NC, Rausch LK, Gatterer H, Burtscher M, Eliasson AH, Pramsohler S. Extreme sports performance for more than a week with severely fractured sleep. Sleep Breath. 2021 Jun;25(2):951-955. doi: 10.1007/s11325-020-02172-4. Epub 2020 Sep 10. PMID: 32909185; PMCID: PMC8195888.
Scheer V, Chandi H, Valero E, Thuany M, Knechtle B, Steinach M. Sleep, Fatigue, and Recovery Profiles of the Longest Solo Unsupported One-Way Polar Ski Journey Across Antarctica. Int J Sports Physiol Perform. 2024 Aug 30;19(11):1328-1333. doi: 10.1123/ijspp.2024-0171. PMID: 39214516.
Scheer V, Chandi H, Valero Burgos E, Thuany M, Knechtle B, Steinach M. Psychological aspects of the longest, solo, unsupported one-way polar ski expedition in Antarctica by a female adventurer. BMJ Mil Health. 2024 Jun 19:e002647. doi: 10.1136/military-2023-002647. Epub ahead of print. PMID: 38897641.
**Duplicate (n=1)**
Bourlois V, Baron P, Hermand E, Elsworth-Edelsten's C, Lemaire C, Hurdiel R. Effet de la sieste sur les temps de réponses et la somnolence perçue lors de l'Ultra Trail du Mont Blanc®, étude pilote observationnelle. Science & Sports.2023. 38(4), 430-434.
**Wrong publication type (n=3)**
Hausswirth C, Louis J, Aubry A, Bonnet G, Duffield R, LE Meur Y. Evidence of disturbed sleep and increased illness in overreached endurance athletes. Med Sci Sports Exerc. 2014;46(5):1036-45. doi: 10.1249/MSS.0000000000000177. PMID: 24091995.
Netzer NC, Rausch LK, Gatterer H, Burtscher M, Eliasson AH, Pramsohler S. Extreme Sport Performance for More than a Week with Power Napping Only. In B66. SRN: CURRENT AND EMERGING TREATMENT THERAPIES TO IMPROVE SLEEP (pp. A3895-A3895). American Thoracic Society. 2019.
Stevenson R. Sleep well, train better. Triathlete. 2011 May 1;(326):148–50.
**Wrong exposure (n=3)**
Filho E, di Fronso S, Forzini F, Murgia M, Agostini T, Bortoli L, Robazza C, Bertollo M. Athletic performance and recovery-stress factors in cycling: An ever changing balance. Eur J Sport Sci. 2015;15(8):671-80. doi: 10.1080/17461391.2015.1048746. Epub 2015 Aug 16. PMID: 26279169.
Jeukendrup AE, Hesselink MK. Overtraining--what do lactate curves tell us? Br J Sports Med. 1994 Dec;28(4):239-40. doi: 10.1136/bjsm.28.4.239. PMID: 7894954; PMCID: PMC1332083.
Saugy J, Place N, Millet GY, Degache F, Schena F, Millet GP. Alterations of Neuromuscular Function after the World's Most Challenging Mountain Ultra-Marathon. PLoS One. 2013 Jun 26;8(6):e65596. doi: 10.1371/journal.pone.0065596. PMID: 23840345; PMCID: PMC3694082.
**Wrong population (n=1)**

Roberts SSH, Teo WP, Aisbett B, Warmington SA. Extended Sleep Maintains Endurance Performance Better than Normal or Restricted Sleep. Med Sci Sports Exerc. 2019 Dec;51(12):2516-2523. doi: 10.1249/MSS.0000000000002071. PMID: 31246714.
**No answer (n=4)**
Doppelmayr et al. (2005) Doppelmayr MM, Finkernagel H, Doppelmayr HI. Changes in cognitive performance during a 216 kilometer, extreme endurance footrace: a descriptive and prospective study. Percept Mot Skills. 2005 Apr;100(2):473-87. doi: 10.2466/pms.100.2.473-487. PMID: 15974358.
Faraguna U, Mastorci F, Banfi T, Giardini G, Pratali L. Impact of a mountain ultra-marathon on sustained attention. High Alt Med Biol. 2014 Jan 1;15(2):A268.
Mougin F, Simon-Rigaud ML, Davenne D, Renaud A, Garnier A, Magnin P. Influence of partial sleep deprivation on athletic performance. Sci Sports. 1990 Jan 1;5(2):83–90.
Sargent C, Martin D, Lastella A, Darwent D, Roach G. Competing for sleep? The amount and quality of sleep obtained by elite cyclists racing in the Australian Tour Down Under. Sleep Biol Rhythms. 2009 Jan 1;7(0):A34.
